# New Advances in General Biomedical Applications of PAMAM Dendrimers

**DOI:** 10.3390/molecules23112849

**Published:** 2018-11-02

**Authors:** Renan Vinicius de Araújo, Soraya da Silva Santos, Elizabeth Igne Ferreira, Jeanine Giarolla

**Affiliations:** Laboratory of Design and Synthesis of Chemotherapeutics Potentially Active in Neglected Diseases (LAPEN), Department of Pharmacy, Faculty of Pharmaceutical Sciences, University of São Paulo—USP, 580–Building 13, São Paulo SP 05508-900, Brazil; renan.arajo@usp.br (R.V.d.A.); soraya.ssantos@yahoo.com.br (S.d.S.S.); elizabeth.igne@gmail.com (E.I.F.)

**Keywords:** PAMAM, dendrimers, drug delivery, gene delivery, nanotechnology

## Abstract

Dendrimers are nanoscopic compounds, which are monodispersed, and they are generally considered as homogeneous. PAMAM (polyamidoamine) was introduced in 1985, by Donald A. Tomalia, as a new class of polymers, named ‘starburst polymers’. This important contribution of Professor Tomalia opened a new research field involving nanotechnological approaches. From then on, many groups have been using PAMAM for diverse applications in many areas, including biomedical applications. The possibility of either linking drugs and bioactive compounds, or entrapping them into the dendrimer frame can improve many relevant biological properties, such as bioavailability, solubility, and selectivity. Directing groups to reach selective delivery in a specific organ is one of the advanced applications of PAMAM. In this review, structural and safety aspects of PAMAM and its derivatives are discussed, and some relevant applications are briefly presented. Emphasis has been given to gene delivery and targeting drugs, as advanced delivery systems using PAMAM and an incentive for its use on neglected diseases are briefly mentioned.

## 1. History

The origin of dendrimers dates back to 1978, when Fritz Vögtle, the then professor of the Kekulé Institut for Organic Chemistry and Biochemistry, reported in his paper “Cascade- and Nonskid-Chain-Like Syntheses of Molecular Cavity Topologie” [1], the newly developed syntheses of many innovative, groundbreaking organic structures. Later on, this led to the development of supramolecular chemistry, including cavitands, cyclophanes, speleands, propeller compounds, and other structures possessing molecular cavity topologies. These iterative-like molecules were the first precursors of poly(propylene imine) dendrimers (PPI) [2].

“A New Class of Polymers: Starburst-Dendritic Macromolecules” described by Prof. Dr. Donald A. Tomalia, in 1985, displays his discussion about the concept, definition, synthesis, and characterization of the “starburst polymers”, a new class of polymers whose building blocks are nowadays referred to as dendrimers. For the first time, polyamidoamine (PAMAM) was presented, as well as its first to seventh synthesized generations [3]. The synthesis from 7 to 11 were also described afterwards, but dendrimers higher than 11, being energetically unstable, cannot be obtained [4].

In a parallel research, Prof. Dr. George R. Newkome reported in his paper “Micelles. Part 1. Cascade Molecules—A New Approach to Micelles”. A (27)-arborol [5], the synthesis and characterization of which he named “unimolecular micelles” or “arborol” (derived from the Latin word *arbor*) possessed the same branching, tree-like architecture of dendrimers. Even though arborols and dendrimers are synonyms, the latter is currently used more often.

## 2. Introduction

Dendrimers comprise a class of nanoscopic compounds, with a well-defined monodispersed and homogeneous molecular structure. Being considered as the fourth and newest class of polymers [6,7,8], these dendrimers differ from the classical oligomers/polymers due to their symmetry, high branching, and maximized terminal functional density [3,9].

**Figure 1:**
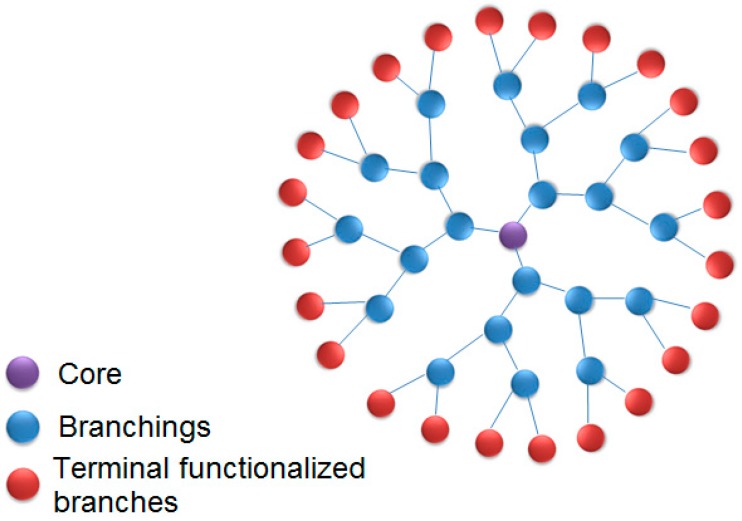
Dendrimers: general structure.

These kinds of molecules are composed of three main components: (1) an initiator multifunctional core, acting as a ‘germination seed’, an anchor point to dendrimer growth; (2) interior layers and inner branches composing the generations; and (3) an outer layer, the terminal functionalized branch, as schematized in Figure 1 [3,10]. The terminal functionality can be modified by adding small molecules, including ions, drugs, and biomolecules. This process can drastically change the physicochemical, reactivity, dynamics, and biological properties of dendrimers.

Considering the most diverse fields on dendrimer applications, such as drug delivery, vaccines, antimicrobials, brain ischemia, and dental glue, the main objective of this paper is to discuss the latest PAMAM advances over the past three years (2016, 2017, and 2018).

## 3. Structure and Synthesis

PAMAM was the first dendrimer that was synthesized and commercialized, and it is the most well-studied and well-characterized class of dendrimers [11]. There are many general approaches for dendrimer synthesis, with convergent [12] and divergent methods [3] being the most commonly used methods. In the last few years, some new methodologies have been developed, such as combined convergent–divergent, click synthesis, hypercores, branched monomers, double exponential, and lego chemistry [7,13,14,15]. 

**Figure 2:**
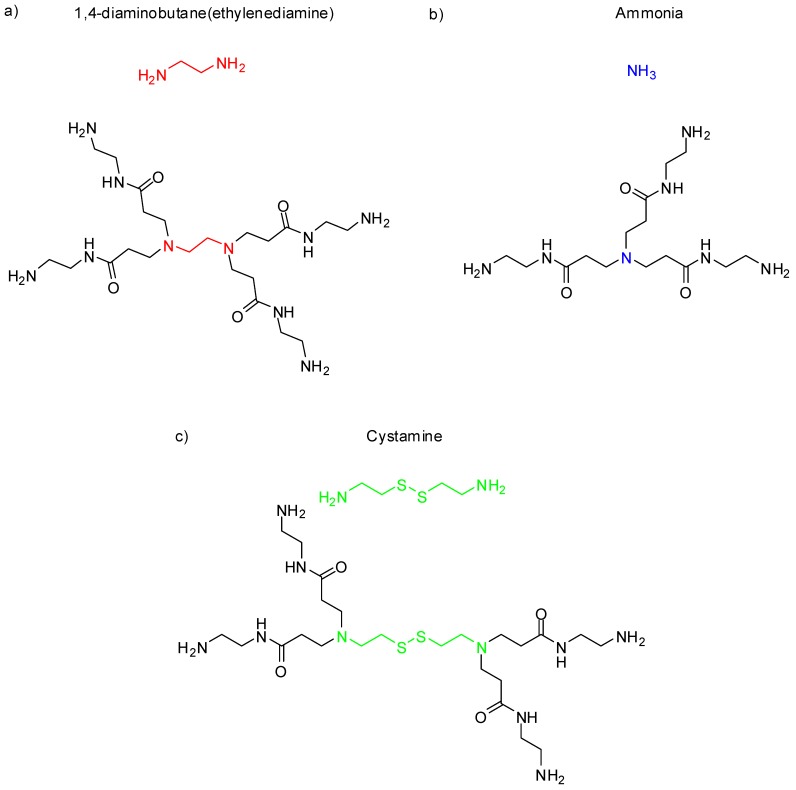
Common cores and G0 polyamidoamine (PAMAM) derivatives of each core.

The PAMAM dendrimer core can be composed of linear chain molecules containing primary amines. The most commonly compounds are ethylenediamine (EDA, four core multiplicity, meaning, therefore, up to four branches), ammonia (three core multiplicity), or cystamine (four core multiplicity), as shown in Figure 2 [7].

**Figure 3:**
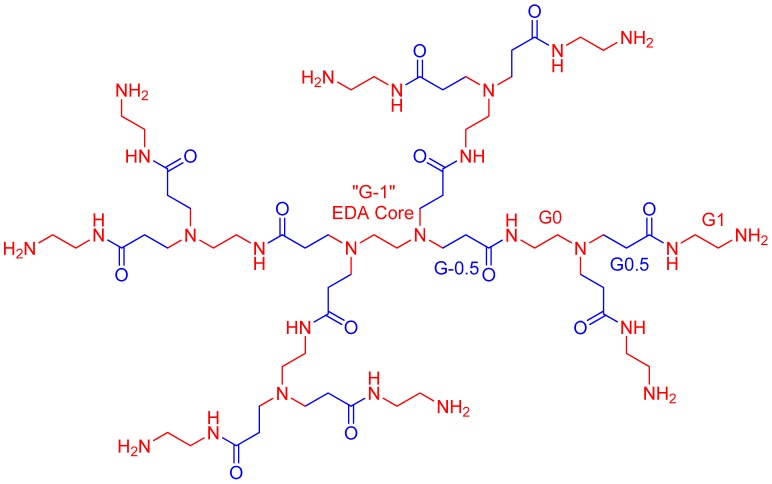
PAMAM generations.

In order to grow further generations, it is necessary to undergo an exhaustive, repetitive, two-step process consisting of (1) Michael addition reactions with an alkyl acrylate, which creates half-generations (i.e., G0.5, G1.5, etc.), composed of terminal ester groups [16,17,18,19]; and (2) in order to obtain full dendrimer generation, ester amidation with an excess of ethylenediamine must be applied. PAMAM-G1, as well as its branching generations, are shown in Figure 3.

The PAMAM molecular weight, the number of atoms, and the terminal primary amine groups increase exponentially for each generation, while the radius increases roughly linearly, by approximately 10 Å per generation [5]. This combination of growth patterns results in a rather interesting property: while low-number generations exhibit almost linear geometries, later generations show more globular-like shapes. The cavities that are intrinsically present in the globular shapes of high PAMAM generations make them suitable agents for encapsulating and adsorbing biomolecules [11,19,20].

The dendritic properties of PAMAM differentiate it from other polymers, since its tree-like architecture enables exponential growth and terminal functionality density [11]. This feature is extensively explored to enhance drug delivery properties, to control release agents [10], and to encapsulate imaging agents [21].

## 4. Toxicity and Safety Concerns

Toxicity and safety are usually the main concerns regarding PAMAM dendrimers. The cytotoxicity is dependent on their concentration, charge, and generation. Cationic derivatives display significant toxicity compared to their neutrally or negatively-charged counterparts, and the cytotoxicity increases along with generation and concentration [22,23,24,25,26,27]. 

PAMAM-G3.5 and PAMAM-G4 dendrimers were evaluated in zebrafish models, an established animal model for a nanoparticle toxicity assay [23,28]. PAMAM-G4 with amine terminal groups showed lethal and sublethal parameters, dose, and exposure time-dependence. PAMAM-G3.5-COOH, on the other hand, was not toxic or deadly at any concentration. Pryor and colleagues employed zebrafish models to analyze the toxicity of PAMAM dendrimers of different generations (G3, G4, G5, and G6) with cationic (–NH2) terminal groups, (G5 and G6) anionic (succinamic acid), and (G6) neutral terminal groups (amidoethanol) [29]. In this study, neutral or anionic dendrimers did not show significant morbidity or mortality at the concentrations tested. On the other hand, positively charged terminal groups induced mortality (lethal effect) and relevant cardiac impacts, as well as pericardial edema (sublethal effect). According to the authors, the terminal group, the number of generations, and the size of the molecules are related to their toxicities in zebrafish models [23,28,29,30,31,32]. For cationic dendrimers, it was verified that more toxicity was present with lower generation and size. These data contrast with findings in cell culture, in which higher-generation dendrimers are more toxic. It is likely that this might have happened because the cationic moieties bind to the cell membrane (negatively charged), and destabilize it, leading to cell lysis [23,33,34].

**Figure 4:**
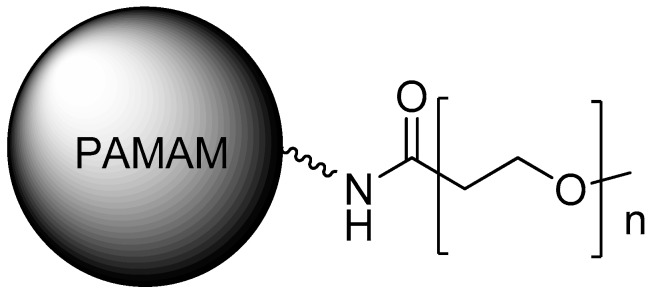
PEGylation of PAMAM is one of the strategies for decreasing its toxicity.

In Caco-2 models, PAMAM dendrimers showed a decrease in cell viability, with generation-dependent toxicity, while higher generations such as PAMAM-G3 and PAMAM-G4 presented lower cell viability than G0, G1, or G2. However, in a similar study performed with L929 mouse fibroblasts, G3 dendrimers did not exhibit significant cytotoxicity up to 1 mg/mL, and no hemolytic effects up to 10 mg/mL, being less cytotoxic and hemolytic than other polycations [24,25,26]. Moreover, the functionalization of the dendrimer surface was assessed regarding the toxicity and immune cell activation, suggesting the possible triggering of inflammatory reactions [35]. Several studies have demonstrated the decrease of PAMAM toxicity through its PEGylation [36] (Figure 4), as reported by Wang et al. (2010) [37].

Najlah et al. [38] (2017) described the use of diethylene glycol (DEG) and lauroyl chains as new surface modifiers to functionalize PAMAM-G0 and PAMAM-G3. The main objective of this work was to decrease the cytotoxicity of naproxen, as well as to improve the pharmacokinetics of the drug profile. The study revealed great enhancement in the transport of naproxen conjugates across Caco-2 cell monolayers, for both lauroyl and DEG derivatives, and for PAMAM-G0 and PAMAM-G3, while PAMAM-G0 conjugates also presented low cytotoxicities. Pyrrolidone as a new surface modifier has also been employed [39,40]. Half-generation anionic PAMAM has been proven to possess very low cytotoxicity, lytic, and hemolytic properties in a broad concentration range. Also, it presented no toxicity in vivo [26,41], this being noteworthy as a promising dendrimer family for future biomedical applications.

PAMAM dendrimers can activate the immune response [42]. This is considering that the positively charged dendrimers may be employed as vaccine carriers, due to their ability to increase cytokine production [36,43]. Regarding PAMAM immunogenicity, the dendrimer was not immunogenic by itself, as it did not induce production against dendrimer-specific antibodies [44,45]. However, the dendrimer conjugation with a protein carrier (albumin to PAMAM-G0 and interleukin-3 to PAMAM-G5 dendrimers) induced the formation of antibodies for dendrimer surface groups, such as amine and oxyamine [45]. Therefore, these studies have indicated the need to conjugate PAMAM dendrimers to a protein carrier for antigenic effect [46].

## 5. Biomedical Applications

### 5.1. Odontology

Wu and coworkers reported the properties of carboxyl-terminated poly(amidoamine) (PAMAM-COOH)-alendronate (ALN) conjugated with (ALN-PAMAM-COOH) on the remineralization of hydroxyapatite on acid-etched enamel, both in vitro and in vivo [47]. Additionally, Wang and coworkers [48] also presented the remineralization properties of PAMAM-G3 for the treatment of dentinal tubule occlusions, which also exhibited great results. Also, Gao and coworkers [49] studied the biomineralization effects of PAMAM-G4. For comparison, sodium fluoride (NaF), being a desensitizing agent, was used as a positive control. Dentine permeability, morphology, and surface deposits were measured, and both samples were submitted to brushing and an acid challenge. The results showed that both PAMAM and NaF reduced dentine permeabilization to significant levels, at 25.1% and 20.7%, respectively. PAMAM also demonstrated good results with creating precipitates on dentine surfaces; it was initially slower than NaF, but then had similar results after 28 days. Moreover, PAMAM induced biomineralization, not only at a superficial level on dentine surfaces, but also on a deeper level, reaching the dentinal tubules. PAMAM still exhibited a stronger resistance to acid challenge, to a greater degree than NaF, and it proved to be a more reliable and stable dentinal tubule occlusion agent. Liang and coworkers [50] also performed similar studies, using a composite with nanoparticles of amorphous calcium phosphate (NACP) and PAMAM, which provided positive results.

Yang Ge and coworkers [51] reported a novel dental adhesive comprising PAMAM-G3 and dimethylaminododecyl methacrylate (DMADDM), which featured not only remineralization, but also anti-caries, and biofilm-regulating properties. The adhesive showed similar bond strengths compared to the control group, lower lactic acid production, and the metabolic activity of biofilms, inhibiting three-species biofilm growth, EPS production, and improved remineralization on dentin, as observed through scanning electron microscopy. PAMAM is regarded as a promising agent for a wide range of applications in the odontology area [52,53,54,55,56,57].

### 5.2. Anti-Atrophics, Analgesics, and Anti-Inflammatories

**Figure 5:**
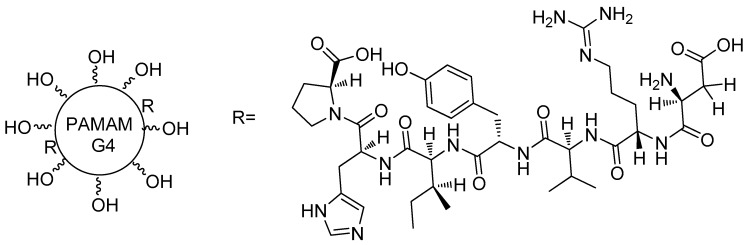
Structure of the PAMAM-G4-OH-Ang-(1-7) compound [58].

PAMAM has also been used as an anti-atrophic agent. Márquez-Miranda and colleagues [58] have reported the application of PAMAM-G4-OH conjugated to angiotensin (1-7) (Ang-(1-7)) (Figure 5) in rats to prevent skeletal atrophy associated to disuse by immobilization. The results demonstrated that PAMAM-Ang (1-7) almost fully recovered muscular fiber diameters and regulated proteins, which in an atrophic state, would be differentially regulated. Those compounds were the pioneers for an injectable formulation, improving the stability of Ang-(1-7) in the human body. The potential of PAMAM for drug delivery was evident, allowing for possibilities for other peptides formulations for disuse-induced skeletal muscle atrophy treatment in humans [59,60,61].

Another use for PAMAM is as an anti-inflammatory and anti-thrombotic agent. Its ability as a nucleic acid scavenging agent has been explored by Lee and colleagues [62], evaluating the extent of the capabilities of both PAMAM-G3 and polyethyleneimine (PEI) as a nucleic acid binding-polymers (NABP). For the experiment, the dendrimer was immobilized on microfiber meshes, as an attempt to attenuate the cytotoxicity of the free dendrimers. The study demonstrated that both dendrimers are able to neutralize damage-associated molecular patterns (DAMPS) and pathogen-associated molecular patterns (PAMP), such as cell-free DNA and RNA. These types of molecules can be identified by specifics toll-like receptors (TLR), and they are known for triggering the immune system. The capacity of the dendrimer for scavenging these molecules can prevent an exacerbated immune response and blood coagulation, which could lead to a thrombotic state. The NABP-immobilized PSMA/polystyrene microfiber mesh (both PEI and PAMAM) reported no cytotoxicity. Overall, both immobilized dendrimers demonstrated great potential for ex vivo treatments, and they can potentially be used in intensive care units, especially on extracorporeal membrane oxygenation, continuous veno-venous hemofiltration (CVVH), and continuous renal replacement therapy, as anti-inflammatory and anti-thrombotic filters for patients with traumas or organ acceptance derived from traumatic injuries. PAMAM-G3 was also applied as a NABP for systemic lupus erythematosus treatment [63]. The findings showed that PAMAM reduced the circulation of soluble autoantibodies, skin inflammation, halted platelet depletion and inflammation-associated organ damage, and generally improved the lupus pathology. This report is believed to be a breakthrough for the application of dendrimers for autoimmune disease therapy.

Dexamethasone conjugated with PAMAM-G4-OH (PAMAM-Dex) was used by Soiberman and colleagues [64] for corneal inflammation treatment, by a cross-linked thiolene click chemistry system and hyaluronic acid. PAMAM-Dex was injected subconjunctivally. The administration of dexamethasone had significant results, such as lower central corneal thickness, better corneal clarity, prevention of high intraocular pressure, and also significant neovascularization reduction, which could be explained by the decrease of macrophage infiltration and pro-inflammatory cytokines. The PAMAM-Dex prodrug depicted a 10-fold effectiveness for the inflammation treatment, when compared to the free drug, and the effects of a single dose remained for two weeks. Therefore, those achievements not only played an important role for steroid-based therapies, but also provided relief and better treatment acceptance by the patient, since the administration was far less frequent, causing much less discomfort.

**Figure 6:**
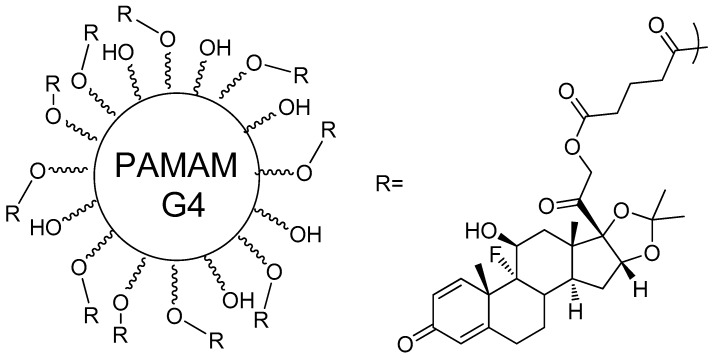
PAMAM-G4 functionalized with 10 molecules of triamcinolone acetonide attached, and glutaric acid as spacer agent [65,68].

PAMAM-G4–OH-triamcinolone acetonide (PAMAM-TA) (Figure 6), characterized by selective uptake towards microglia cells, was synthesized and administered intrathecally into the spinal cords of mice by Kim et al. [65] to evaluate its effect as an anti-inflammatory and analgesic [65,66,67]. When tested upon microglia cells in mice, PAMAM-TA prevented the upregulation of proinflammatory cytokines upon stimulation with lipoteichoic acid at levels as high as 90%. The compound also relieved nerve injury-induced neuropathic pain, due to the activation of spinal cord microglia, lowering Nox2, IL-1β, TNF-α, and IL-6 upregulation by up to 90%, when compared to the control group. This measurement suggests that PAMAM-TA could also have pain relieving effects, and the hypothesis was addressed through a van Frey test, which confirmed its strong analgesic effects in mice. The effects of a single dose lasted for up to three days and showed no cytotoxicity. The PAMAM-conjugated TA can represent a great improvement to free TA, as it prevents direct injections to the central nervous system (CNS), considering that these are very risky, and it also prevents the high levels of steroids, which could cause neurotoxicity. A previous study by Kambhampati [68] also reported on the use of the same compound for the treatment of retinal epithelial pigment cells, where the PAMAM-TA compound presented a better inhibition of VEGF production on the hypoxic cells, even with a 100-fold lower concentration than free TA. Both neuroinflammation and VEGF production are keys to diseases such as diabetic macular edema, proliferative diabetic retinopathy, and exudative age-related macular degeneration [69,70].

### 5.3. Targeting Dendrimers

**Figure 7:**
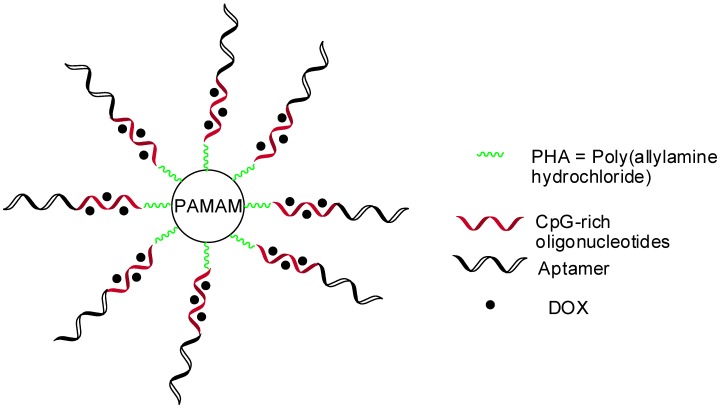
Aptamer-PAMAM dendrimers coupled with CpG-rich oligonucleotides loading doxorrubicin–chemotherapeutic drug (DOX) [71].

Nucleic acid aptamers have received prominence as drug carriers, due to their high affinity to specific ligands, their chemical flexibility, and their tissue permeability. Different aptamer–drug complex or nanoparticles systems were obtained for several applications, such as toxins, peptides, chemotherapeutics, oligonucleotides, and aptamer-mediated drug delivery, cancer treatment, neurological, and immunological diseases. Aptamer-PAMAM dendrimers were developed for targeting in chemo-immunotherapy using aptamers coupled to PAMAM through CpG-rich oligonucleotides carrying, also, DOX (doxorrubicin–chemotherapeutic drug) (Figure 7) [71].

**Figure 8:**
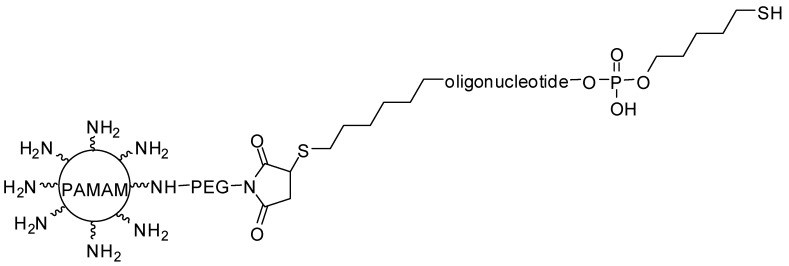
Smart AS1411 aptamer-functionalized/PAMAM dendrimers as nanocarriers for targeting drug delivery for gastric cancer [74].

PAMAM succinamic acid, in another investigation, was conjugated to oligodeoxynucleotides on the surface, resulting in single-stranded oligodeoxynucleotide-conjugated dendrimers (sONT-DENs). Anti-prostate-specific membrane antigen (PSMA) aptamers were functionalized with the sONT-DENs, resulting double-stranded aptamer-sONT-DEN derivatives, and then DOX was encapsulated. This complex displayed antitumor activity and in vivo specificity in prostate tumor models [72]. PEGylated PAMAM has also been hybridized with anti-nucleolin aptamer and 5-fluorouracil (5-FU-anticancer drug), revealing 5-FU-specific accumulation in target tumor cells [73]. Additionally, three different aptamers (MUC-1, AS1411, and ATP) were conjugated to dendrimers for targeting delivery of epirubicin (chemotherapeutic agent). The derivative dendrimers containing AS1411 aptamer is exhibited in Figure 8 [74]. These compounds were internalized into tumor cells, and presented acceptable cytotoxicity in vitro, and inhibition of tumor growth in vivo [73].

Folic acid is the directing group of folate receptors, and it is widely used as targeting nanocarriers for anticancer drugs. PAMAM dendrimers were functionalized with folic acid and isothiocyanate (fluorescein agent), and DOX was loaded. This compound was planned to act simultaneously as a drug-targeted and pH-responsive system, therefore carrying DOX into tumor cells. The findings showed high affinity to folate receptors and a therapeutic efficacy for the dendrimers tested [75,76].

The use of stem cells and PAMAM were also investigated. Dendrimer surfaces were coated with adhesion molecules that were selectively expressed on the cellular endothelium, aimed at reaching diseased tissues such as bone marrow cells. In this study, the authors coated the surface of stem cells with PAMAM, which were previously coupled with adhesion moieties. The latter molecules are targeting groups for surgically created cutaneous and corneal wounds. These derivatives showed advantages in relation to ordinary bone marrow cell transplantation in mouse models, especially for wound healing and neovascularization. Also, they observed the specific delivery of coated stem cells to cutaneous wound tissues and injured corneas, without uptake in common organs such as the lung, liver, and spleen. Thus, the planned compounds proved to be specific, and part of a biocompatible strategy that is used to increase the efficiency of drug targeting for regenerative diseases. This cell coating technology can mediate cell–cell interactions and tissue-targeted cell delivery for wound healing, as well as for non-toxicity in vivo (murine models) and in vitro (human cells) [77].

### 5.4. Gene Delivery

Alternatives to viral-mediated gene delivery for gene therapy have been actively studied recently [78]. There is a growing concern related to the use of virus for this purpose, since it shows a lack of specificity, a low transfection rate, immunogenicity, toxicity, or even oncogenicity [79]. In this context, dendrimers are being largely used as an effective non-viral mediated gene delivery system. The amine functionalities of PAMAM, as well as the surface groups presented in PEI, can bind effectively to nucleic acids in physiological pH, due to polycationic nature of these compounds [80]. They offer advantages over other polymers, such as the control of physicochemical and pharmacokinetic properties, and the possibility of addition of protecting or directing groups. The transfection efficacy of PAMAM dendrimers tends to increase with generation, reaching a stable transfection rate in the eighth generation [81].

Different strategies have been used to improve the properties of PAMAM as a non-viral mediated gene delivery agent. Conjugation of group II chaperonin thermosome (THS) in PAMAM-G4 was tested by the group of Nussbaumer [82]. This compound was considered to be a new architecture for gene delivery, carrying KIF11- and GAPDH-silencing interfering RNAS (siRNAS) to cancer cells with good results, significantly inhibiting the proliferation of those cells.

**Figure 9:**
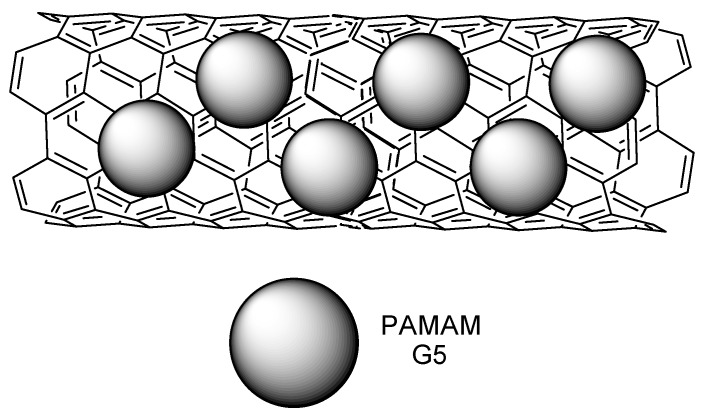
Schematic representation of the proposed binding mode of PAMAM on carbon nanotubes (CNT) [83].

Carbon nanotubes (CNT) were used as another platform for binding PAMAM and PEI for delivering microRNAS (miRNAS) [83]. Different sizes of CNT were used, including biodimensional sheets (bucky papers (BP)) and the corresponding molecules, which are effective for transfection in different ways (BP, for example, can act as a ‘nanoneedle’ to pierce the cell). The CNT strategy [83] was then be applied by Masotti and coworkers [84], employing PAMAM and PEI coated with CNT, which was conjugated with miR-503 oligonucleotides, aiming at the regulation of angiogenesis in endothelial cells. On the other hand, miR-503, whose target is named CDC25A, is overexpressed on diabetes mellitus, and also the gene that is responsible for endothelial cells proliferation [85]. The study reported not only 99% of PAMAM-CNT-miR-503 system transfection efficacy, but also highly efficient miRNA release in endothelial cells, being therefore able to regulate the cell proliferation population. The stability of mir-503 towards RNAse was also greatly improved. Finally, the number of vessels on a sponge model subcutaneous implant on mice treated with PAMAM-CNT-miR-503 was significantly reduced when compared to both the control and free miR-503 (Figure 9).

A study regarding new method of delivering the H5-DNA vaccine against avian influenza was performed by Bahadoran [86]. Using a complex system with the H5-Green Fluorescent Protein gene cloned into the expression vector pBud, Bahadoran designed a system composed of pBud-H5-GFP and the interferon-regulatory factor (IRF)3. This pBud-H5-GFP system was then conjugated into a transactivator of transcription (TAT) bound with PAMAM (PAMAM-TAT). The results demonstrated low toxicity and good skin permeation. The cellular uptake of the pBud-H5-GFP-IRF3 was better when conjugated to PAMAM-TAT, compared to the free dendrimer. The introduction of the IRF3 group improved the immune response, a common problem faced with some DNA vaccines [87].

The effects of a double suicide gene, cytosine deaminase (CD) in combination with thymidine kinase (TK)—conjugated with PAMAM-G5, as anti-scarring agents on human Tenon’s capsule fibroblasts—were studied by Yang and colleagues [88]. In therapy, there are simultaneous administrations of ganciclovir (GCV) and 5-fluorocytosine (5-FC), aiming at the prevention of infection in the post-operative period of glaucoma filtering surgery. The TK gene can turn GCV into its phosphorylated form, and the CD gene can transform 5-FC into 5-fluoruracyl. Both transformed compounds are highly cytotoxic to the cells that express these genes, with low or no cytotoxicity to human cells [89,90]. The cell viability was greatly reduced when both 5-FC and GCV were administered to CV- and TK-transfected cells. Although the low transfection efficiency of PAMAM in the assay, the “bystander effect” [91] occurred, killing the non-transfected cell population nearby transfected cell populations, enhancing the efficacy of the treatment.

**Figure 10:**
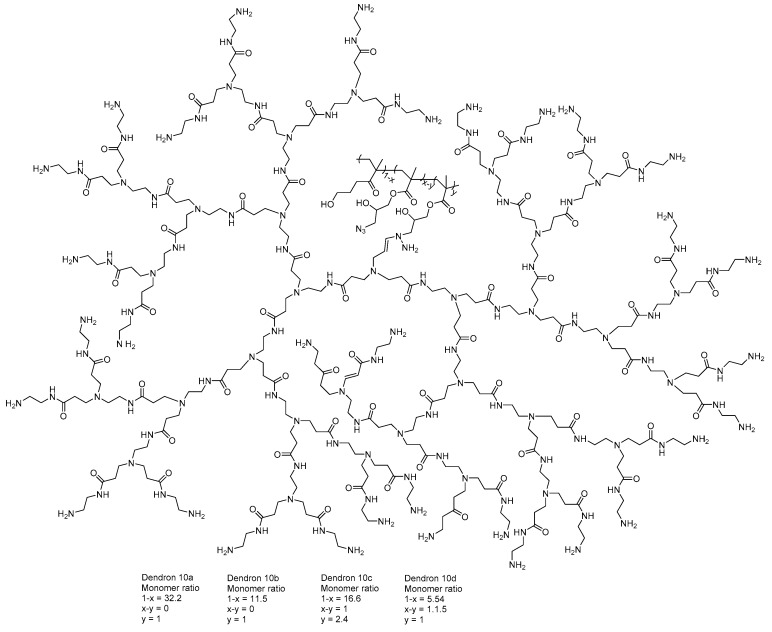
PAMAM-G5 functionalized with linear copolymers for gene delivery as CRISPR (clustered regularly interspaced short palindromic repeats) [92].

Kretzmann et al. [92] designed a flexible dendritic polymer to solve the delivery difficulties related to genomic engineering. Linear dendronized polymers are controllable and they represent synthetic platforms, providing an efficient and atoxic agent for delivering precise gene editing tools, such as CRISPR (clustered regularly interspaced short palindromic repeats) systems and TALE (transcription activator-like effectors) proteins. The authors above reported that redesigned dendrimer architecture may solve the packaging ability and toxicity troubles that are related to higher level of generation (Figure 10).

Gold–PAMAM dendrimers (AuPAMAM) were designed to transfect cells, proving to be quite efficient in this aspect. The respective compound was analyzed in two cell lines: (1) a “hard to transfect” CT26 cell line; and (2) an “easy to transfect” SK-BR3 cell line. Many intracellular transport mechanisms, which affect the gene therapy, unknown, presenting great barriers to the planning of gene delivery carriers. Figueroa et al. [93] investigated the intracellular processes that may explain cell-to-cell variations, as well as vector-to-vector distinctions in the gene transfectability of AuPAMAM. The findings demonstrated higher transfection in the SK-BR3 cell line than in CT26. The latter cells presented greater hindrance, which impairs the internalization DNA/vector complexes in comparison to SK-BR3 cells. This investigation was the first step to identifying and designing more effective application-specific non-viral carriers. 

**Figure 11:**
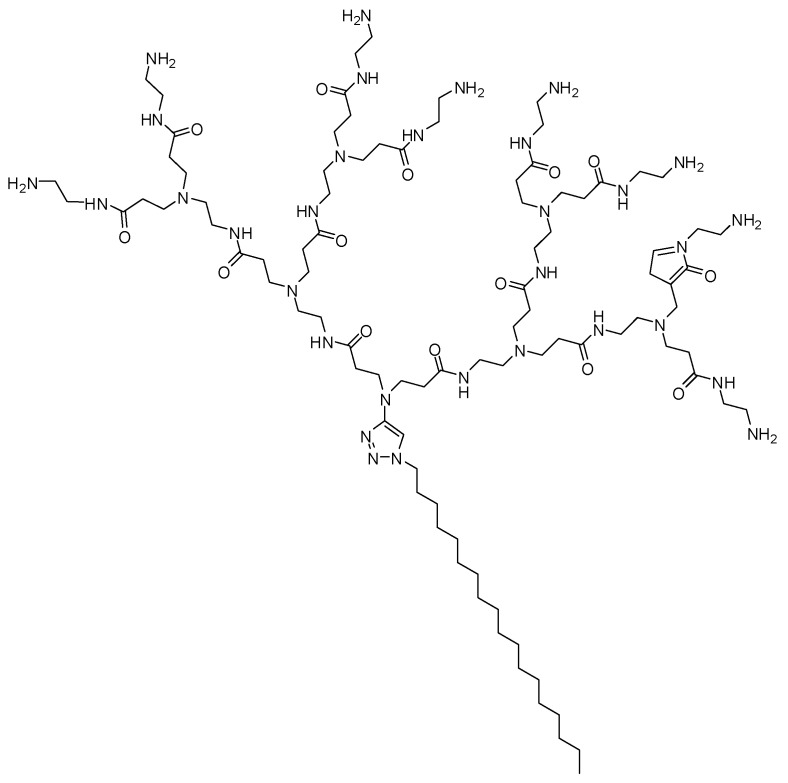
Amphiphilic promising dendron for small interfering RNA (siRNA) delivery [94].

In another study, Chen and co-workers [94] developed self-assembling PAMAM dendrimers for small interfering RNA (siRNA) delivery. These compounds consisted of small amphiphilic dendrons in dendrimer micelles, showing a structural definition equivalent to common high dendrimer generations. This research group has been studying PAMAM as nanovectors for siRNA delivery in vitro and in vivo, and currently, one of them is being tested in clinical trials [95,96,97,98,99,100,101]. In this investigation, the authors synthesized amphiphilic molecules composed of a hydrophilic PAMAM dendron head, and a hydrophobic portion containing a linear hydrocarbon chain of variable length (Figure 11), and their performance in siRNA delivery was affected by the equilibrium of the hydrophobic and hydrophilic components. In addition, these self-assembling dendrons, which presented no toxicity, reached gene silencing in highly refractory human hematopoietic CD34 stem cells, which might be explored for future biomedical applications [94].

**Figure 12:**
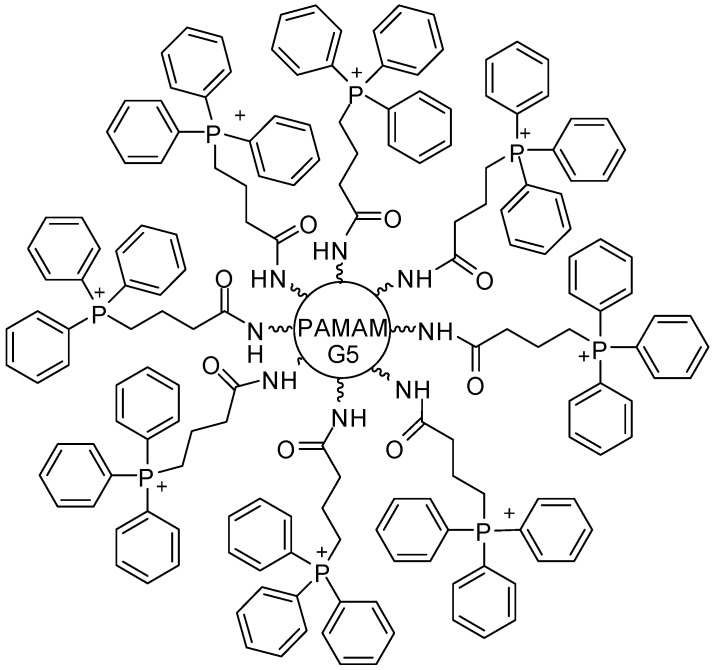
Dendrimer E9-2 with a high ability of gene transfection in a stem cell model [102].

Liu and colleagues [102] published a library of surface-engineered dendrimers, which can be applied as siRNA carriers. Structure–activity relationship (SAR) studies demonstrated that hydrophobic ligands—such as aliphatic chains, aromatic rings, fluorine, and bromine atoms—are fundamental for polymers, since they may provide high levels of gene silencing efficacy. Among the obtained compounds, the intermediate E9-2 (Figure 12) displayed higher transfection in stem cells than commercial lipid carriers, as Lipo 2000. It is important to highlight that the derivatives were able to deliver several siRNA into different cell lines, with a minimum level of cytotoxicity. Currently, SAR findings provided the development of a second-generation library of dendrimers with high gene transfection activity, contributing to the rational design of further potent siRNA nanocarriers. 

The surfaces of baculoviruses was modified with a PAMAM dendrimer, resulting in a kind of compound that was capable of efficiently loading the *VEGF* transgene in transduced human adipose-derived stem cells (hASCs), which can overexpress pro-angiogenic genes. According to the authors, this derivative presented enhanced transduction efficiency based on dendrimer features. Furthermore, assays with transduced hASCs into a murine myocardial infarction model showed vascularization increase and cardiac function improvement, when compared with control therapy containing unmodified hASCs [103,104]. The same research group developed PAMAM–baculovirus complexes that were microencapsulated in poly(glycolic-co-lactic acid) (PLGA) to overcome plasma inactivation and to extend gene delivery. The stent was coated with the respective microcapsules, and it was implanted in canine femoral arteries. The pro-angiogenic effects of the VEGF-mediated baculovirus treatment were observed with regard to endothelial regeneration in injured regions four months after its application [105].

The PAMAM dendrimer was also coated with recombinant baculovirus to stimulate the overexpression of VEGF (vascular endothelial growth factor—a protein that is responsible for stimulating of the formation of blood vessels). This approach could be employed to repair damage in cardiac tissue [106]. 

Dendrimers as a non-virus mediated gene delivery system have also been used extensively in the fields of neuroscience [79,107], cancer [108,109], and tissue reparation [110], among others [111].

### 5.5. Oral Delivery

PAMAM dendrimers have been extensively studied as an oral drug delivery vehicle, since it presents the ability to cross intestinal epithelial barriers. Several investigations have been published, aiming to understand and/or improve pharmacokinetic parameters. The major concern is that PAMAM toxicity and biocompatibility are mainly related to the chemical groups that are present on the surface [112].

Propranolol (a poorly soluble drug) was conjugated to PAMAM-G3 and PAMAM-G3–lauroyl dendrimers, using a chloroacetyl spacer. The respective prodrug increased their drug water solubilities, and also enhanced its apical-to-basolateral transport across Caco-2 cell lines [113]. In vivo oral absorption was similarly analyzed using PAMAM-G3 functionalized with DOX. The findings revealed a 300-fold increase in the oral bioavailability of the prodrug when compared to the free drug [114]. 

**Figure 13:**
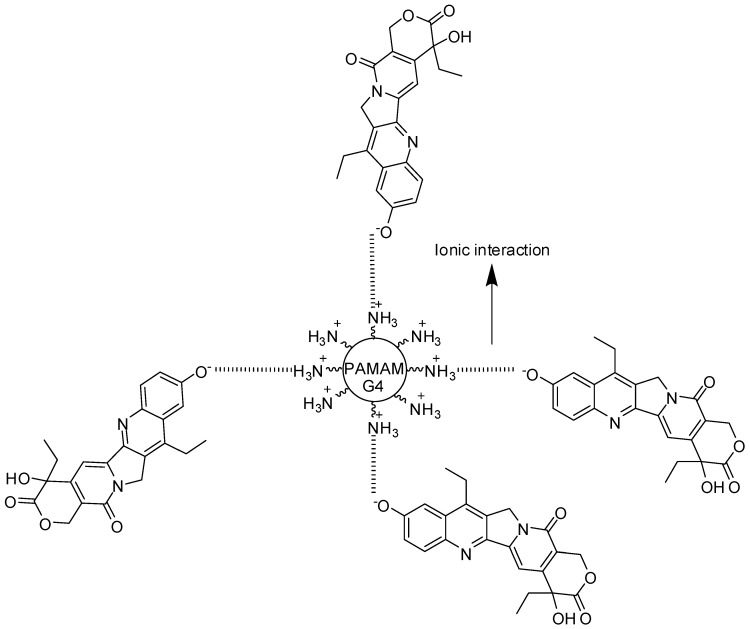
A PAMAM dendrimer complexed to 7-ethyl-10-hydroxy-camptothecin [115].

Kolhatkar and co-workers [115] developed PAMAM derivatives complexed to 7-ethyl-10-hydroxy-camptothecin (SN-38—an anticancer drug). These compounds presented up to a 10-fold higher permeability, as well as an increase of 100-fold cellular uptake when compared with free SN-38 (Figure 13). Camptothecin was also formulated and co-delivered with cationic PAMAM-G4 and PAMAM-G3.5–COOH. Both dendrimers exhibited an increase of 2- to 3-fold intestinal absorption of camptothecin in vivo [116]. As a conclusion, PAMAM dendrimers can be applied to enhance the intestinal permeability of drugs with poor oral bioavailability, as well as targeting drug delivery [112].

### 5.6. Pulmonary Biodistribution

PAMAM dendrimers have been investigated as pulmonary drug delivery agents through oral inhalation. PAMAM-G3 and its PEGylated counterpart were evaluated for lung cellular biodistribution. The compound presents a peak of concentration in systemic circulation within a few hours after pulmonary delivery in the presence or absence of PEG, presenting positive or negative charges, even with different sizes. However, highly PEGylated dendrimers demonstrated the highest peak plasma concentration upon pulmonary delivery. These results can be useful for designing strategies regarding dendrimer-based pulmonary drug delivery [117]. 

Anionic PAMAM dendrimers and dextran probes, two polymers containing similar molecular sizes, were analyzed by endocytic uptake in lung tissues. Both compounds were passively absorbed in the intact lung. However, the dendrimer compound showed a slower absorption rate when it was compared to dextran. Moreover, anionic PAMAM presented lung biocompatibility and quick uptake into the pulmonary epithelium, and this extended the transportation in the lung. These results therefore support the use of delivery systems of inhaled PAMAM-based drugs for its controlled release to the lung [118].

### 5.7. Antimicrobial Activity

**Figure 14:**
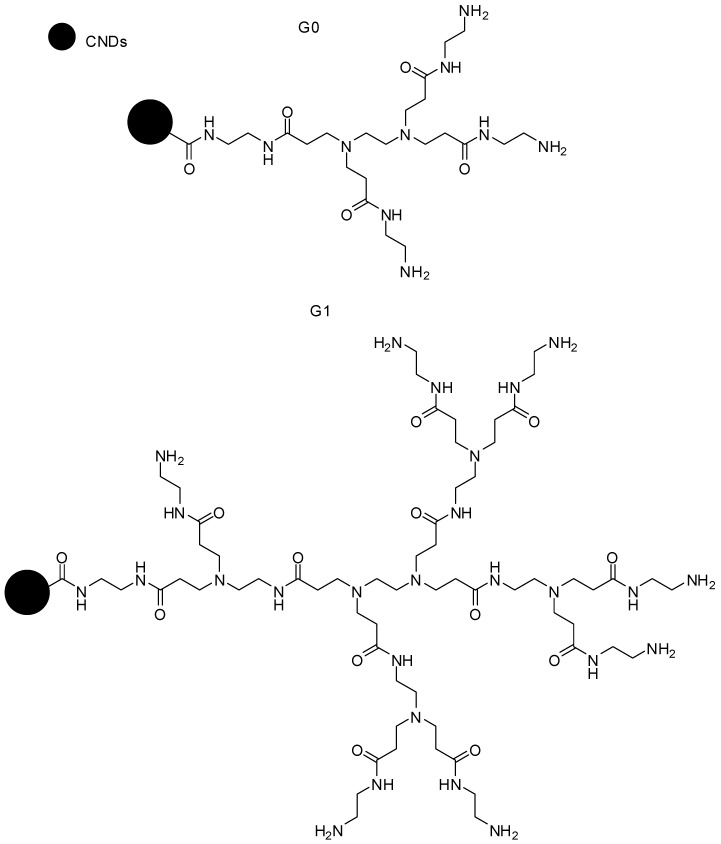
PAMAM dendrimer conjugated to CNDs (carbon nanodots) [119].

Carbon nanodots (CND), obtained from starch and other carbon sources, have been used for different biomedical applications, as they present low toxicity, as well as fluorescent features. PAMAM dendrimers (generations greater than three, named as G3) are large polycationic molecules, demonstrating antibacterial properties, while the lower-numbered generations did not show a degree of antimicrobial efficacy. Studies reported that PAMAM can also damage the integrity of microbial membranes, which possess an overall anionic charge. Moreover, the respective compounds can improve the cell uptake of antibiotics into the bacterium, consequently showing synergistic effects. In this context, CNDs could act as a molecular scaffold for grafting small polycationic amines, since they exhibit high cationic surface density, therefore improving the antimicrobial action. Ngu-Schwemlein and colleagues [119] conjugated PAMAM-G0 and PAMAM-G1 dendrimers (Figure 14) containing CNDs as molecular scaffolds, to increase their aminated cationic concentration, and hence, evaluated their antimicrobial activity. Furthermore, these PAMAM dendrimers conjugated to CNDs were assessed in association with tetracycline or colistin, presenting antibacterial synergic action.

PAMAM-G7 was assayed against Gram-negative and Gram-positive bacteria. The dendrimer was potentially able to inhibit the growth of both kinds of microbia, and their cytotoxicity was dependent on the exposure time and the concentration. Although more studies are required, this investigation suggested that PAMAM-G7 could be a potential antimicrobial agent. According to the authors, the antibacterial property of dendrimers is probably mediated through the disruption of the bacterial membrane by terminal amine groups. These functional groups are taken up onto bacterial cell surfaces, and then diffused through the cell wall. Thus, dendrimers interact with the cytoplasmic membrane, resulting in its disruption and disintegration, which results in the release of electrolytes, such as potassium and phosphate ions—and nucleic acids such as DNA and RNA—from the cell [120].

Similar investigation was developed by Rastegar and co-workers [121]. The authors synthesized PAMAM-G6 and evaluated its antibacterial properties against Gram-negative bacteria (*P. aeruginosa* and *E. coli*, for example) and Gram-positive bacteria (*S. aureus* and *B. subtilis*), which were isolated from different clinical samples, as well as from microorganisms standard strains. The dendrimer exhibited a good response regarding removal of microorganisms in both conditions, being an effective antimicrobial agent. According to the authors, the mechanism of action was the induction of autophagy and the decrease of *P. aeruginosa* infection and growth. Moreover, it displayed mucolytic activities, indicating its potential in recovering *P. aeruginosa* cystic fibrosis lung disease induction.

Several studies demonstrated the ability of the PAMAM dendrimer to improve antifungal activity as consequence of increased drug solubility in water [122,123]. Winnicka et al. [124] reported the water solubility increase of clotrimazole, which, consequently enhanced its antifungal action against different *Candida* strains. In another investigation, hydrogels of polyacrylic acid-containing clotrimazole, PAMAM-G2 and PAMAM-G3 (–NH_2_ or –OH terminated groups) induced bioadhesive features and viscosity [125]. The same research group formulated a mixture containing ketoconazole (hydrophobic drug) and PAMAM-G2 (–NH_2_ or –OH terminal groups), which was demonstrated to be 16 times more potent against *Candida* when compared to the free drug [126].

PAMAM dendrimers from different generations (G1–G3) were explored as agents for improving amphotericin B water solubility. The findings showed great water solubility regarding the higher generations and the concentration. Also, the complexed amphotericin–dendrimer showed sustained release in vitro [127].

In another antimicrobial approach, PAMAM-G5 dendrimers were combined to SIRT-1 (an enzyme involved in HIV-1 replication) inhibitor (HR-73) for brain HIV-1 infection. The results showed an increase of SIRT-1 inhibition, which could act in HIV reactivation from latent reservoirs. All findings therefore suggested the importance of the association between the drug and the dendrimer, since its ability to deliver anti-retroviral drugs could improve the drugs’ pharmacokinetics [128].

### 5.8. Other Applications

**Figure 15:**
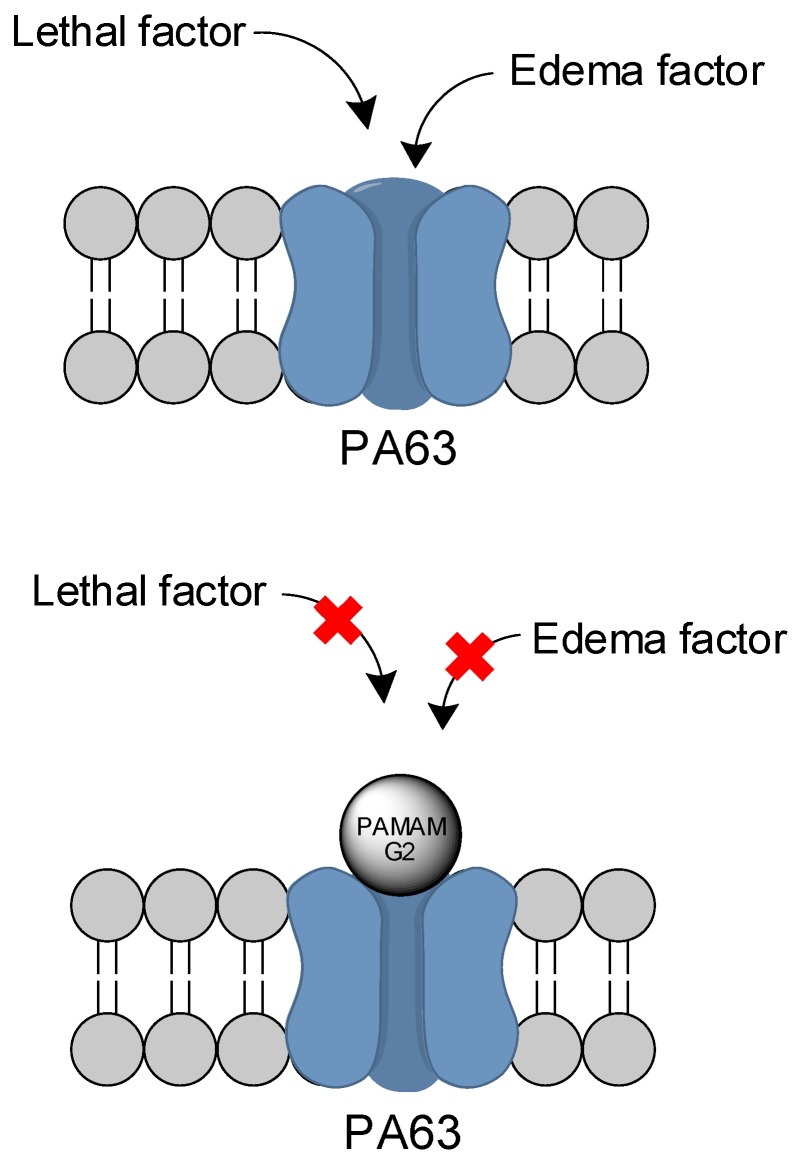
Blockage of the PA63 channel by PAMAM compounds [129].

Several dendrimers of PAMAM-G2 and PAMAM-G3 containing anionic, neutral, and/or cationic surfaces were studied by Yamini and coworkers [129] to measure their effects on the protective antigen (PA) blockage channel of the anthrax toxin. PA, more specifically in an 83 kDa monomer peptide (PA83) present in the anthrax toxin, which is cleaved to PA63 [130]. The latter peptide can then oligomerize into a ring-shaped heptamer, facilitating the uptake of lethal and edema factors into the host cells. Also, it is present in the anthrax toxin, leading ultimately to cell death [131]. PAMAM can inhibit PA63 activity by blocking the lumen channels that are negatively charged, as illustrated in Figure 15. Cationic PAMAM compounds performed better, with an IC_50_ of 7.2 ± 4.7 nM for the best PAMAM-G2–NH_2_ compound (in contrast to 14 mM for the anionic derivative). A dendron of cationic PAMAM-G3 was also able to diminish the toxicity issues, possessing an IC_50_ of 16.4 ± 4.0 nM. PAMAM-G2, containing 12 hydroxyl functionalities and four terminal amino groups (PAMAM-G2, 75% OH, 25% NH_2_) presented an IC_50_ of 122 ± 35 nM. All cationic PAMAM compounds also performed well in the kinetics studies, presenting a decent residence time and dissociation constant. The inhibition of PA63 activity can potentially prevent infected cell death, and its efficacy can be evaluated in further investigations.

Zhao and coworker [132] employed PAMAM–polyvalerolactone–poly(ethylene glycol) (PAMAM–PVL–PEG) to design a unimolecular micelle (unimNP), aiming to address the problem of retinal ganglion cell (RGC) loss on glaucoma. This unimNP was conjugated to cholera toxin B (CTB) domain to make the micelles selective towards the RGC. Dehydroepiandrosterone (DHEA) was used as agonist for the Sigma-1 receptor (S1R), a protein receptor that proved to be RGC-protective [133,134]. In vitro studies demonstrated sustained release of DHEA for periods of up to two months. On the other hand, in in vivo studies with mice injected with *N*-methyl-D-aspartate, which can induce RGC death, unimNP with CTB were able to protect RGC from cell death for periods of up to 14 days, and attenuate oxidative stress and the activation of microglia/macroglia. S1R has shown increasing evidence of playing a major role in a plethora of neurological diseases—such as depression, anxiety, schizophrenia, Parkinson’s disease, Alzheimer’s disease, amyotrophic lateral sclerosis, stroke, and others [135,136]—showcasing the importance of a sustained release platform for targeting the respective receptor.

A liver-cell spheroid shaped, aggregated in a human hematopoietic stem cell culture, was achieved by Chen and coworkers [137] using PAMAM conjugated with integrin ligand, arginine-glycine-aspartic acid (RGD), and PEG, resulting in a PAMAM–RGD–PEG compound. PAMAM–RGD–PEG promoted the hepatocytes growth in 3D spheres, as this condition mimics the cell’s natural microenvironment. Additionally, it also helped to maintain cells in a healthy and functional state, greatly decreased the presence of ROS, promoted cell proliferation, and led to higher concentrations of albumin and urea. Furthermore, it also leads to a higher expression of urea-dependent enzymes Arg1 and OTC, enhancing the basic functions of the cells. These cells could potentially represent a great advance for bioartificial liver systems, being extracorporeal detoxification systems that are useful for liver failures [138]. 

PAMAM dendrimers were applied to control diabetes, since it is one of the leading causes of death worldwide [139]. Zhang and co-workers [140] then designed an advanced glucose-sensitive system, consisting of a smart microgel derivative from PAMAM-G1 dendrimers [141,142]. 

**Figure 16:**
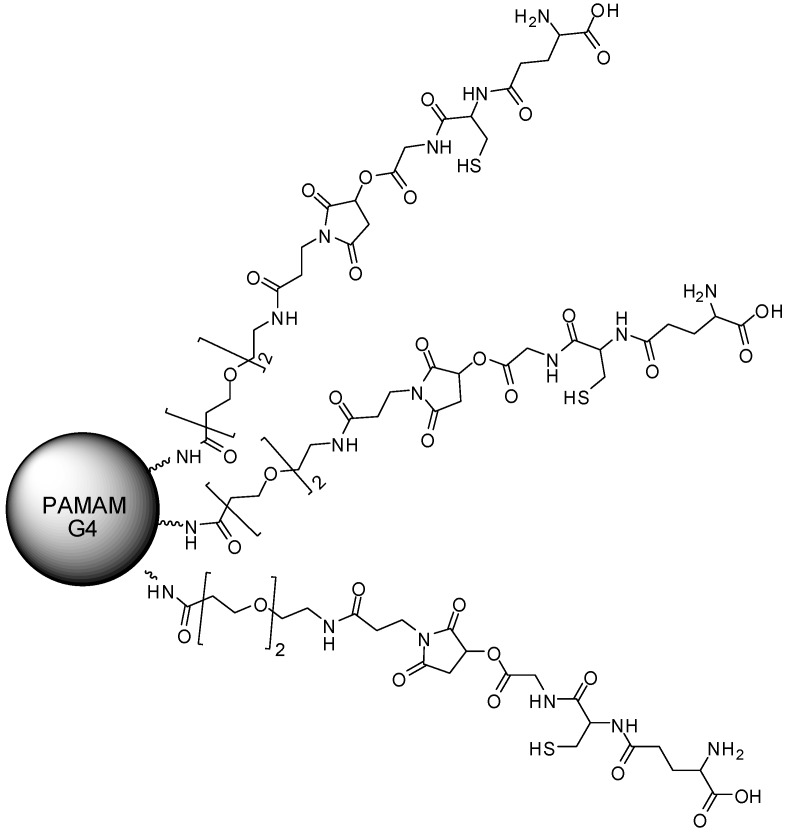
The PAMAM–poly(ethylene glycol) (PEG)–glutathione (GSH) compound [142].

Parkinson’s disease (PD) was assessed using a PAMAM-G4 conjugated to glutathione (PAMAM–GSH) (Figure 16), aiming to deliver glutathione into PC12 cells [143]. Glutathione is a tripeptide that plays an important antioxidant and antiapoptotic role in the brain, whose depletion has already been correlated to PD [144,145]. PAMAM–GSH was able to lower intracellular levels of reactive oxygen species, and to reduce the levels of cleaved caspase-3 in the assays, achieving 10% of their original concentrations. Curiously, these antioxidative and antiapoptotic properties were significantly higher when PAMAM–GSH was at low concentrations. PAMAM–GSH also demonstrated a lower cytotoxicity than the PAMAM-G4 itself. Other strategies for the treatment of PD with PAMAM were also explored in the past [146,147]. There are also reports on PAMAM being applied to Alzheimer’s disease and other prionic diseases [148,149,150].

Nitric oxide (NO)—an important free radical gas—exhibits actions such as antimicrobial action, muscle relaxant, vasodilation, and the stimulation of growth factor [151]. However, NO is unstable under different conditions, especially in biological environments. In this context, Stasko et al. [152] designed a modified PAMAM dendrimer as a NO delivery carrier. This vehicle can store up to 2 μmol NO/mg dendrimer, and releases NO when triggered with copper (II) or light. Other examples of PAMAM dendrimers functionalized with NO donors groups (*S*-nitrosothiols, *N*-acetylpenicillamine and *N*-acetylcysteine) have been described [153,154].

**Figure 17:**
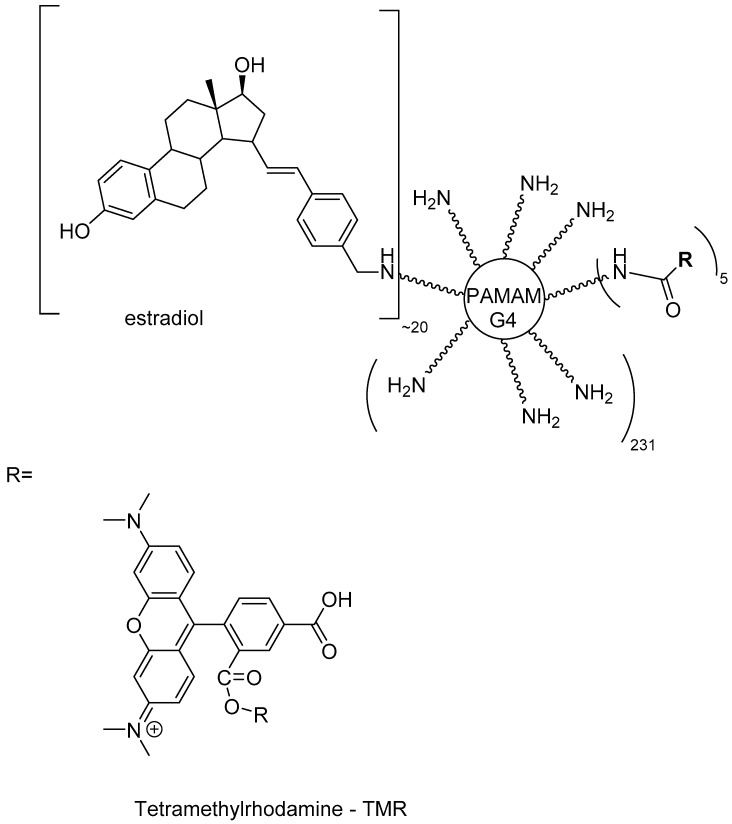
A PAMAM dendrimer conjugated to estradiol [151].

In a different approach, estradiol was conjugated to a positively charged PAMAM dendrimer through a hydrolytically stable bond. Also, the dendrimer was coupled to the fluorescent reagent tetramethylrhodamine, which provides a convenient way to improve intracellular visualization (Figure 17). This compound was able to bind to the estrogen receptor with a similar affinity as free estradiol. Even though it cannot be taken up into the nucleus, it can still stimulate gene expression. According to the authors, this derivative can be applied to mimic the protective effects of estradiol on the cardiovascular system in mice [155].

**Figure 18:**
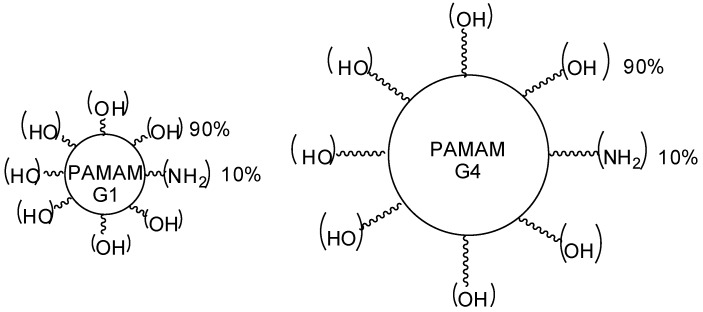
PAMAM-G1 and PAMAM-G4 dendrimers containing mixed surface groups, neutral (–OH—90%) and cationic (–NH_2_—10%) moieties [152].

Srinageshwar and colleagues [156] evaluated novel mixed surface PAMAM dendrimers that are able to cross the blood–brain barrier when administered via the carotid artery in mice. In this investigation, there was a comparison between PAMAM dendrimers composed of neutral (–OH) and cationic (–NH_2_) groups on the surface, called the mixed cationic surface (–NH_2_) (Figure 18). According to the authors, mixed surface compounds were able to cross the blood–brain barrier (BBB) when injected systemically. Thus, they could reach the brain cells, indicating that they are a potential CNS drug delivery system. In addition, these compounds demonstrated reduced toxicities.

## 6. Concluding Remarks

After more than three decades of dendrimer discovery and the introduction of PAMAM, this area has advanced significantly, and newly-derived molecular architectures have arisen from this starting point. However, PAMAM continues to be the dendrimer basis for many applications, such as those showcased in this review. It is worth noting the versatility of PAMAM as an important carrier for drug delivery and biomedical applications. Studies with many therapeutic classes have been developed over the years. Drugs that are either covalently linked (prodrugs) or adsorbed to PAMAM branches have been assayed, with most of them being chemotherapeutic agents, such as anticancer agents and antimicrobials. In the era of gene therapy, the role of PAMAM as a non-viral mediated gene delivery system must be emphasized. Many examples have been briefly described—such as dendrimers of silencing RNA and precise gene editing tools, such as CRISPR—whose use deserves increasing interest. Notwithstanding, some areas should stimulate interest in using dendrimers as PAMAMs. Neglected diseases, especially those that have been nearly forgotten, need more effort in studies that are purposed towards better chemotherapeutic agents. The therapeutic arsenal against most of them is very scarce or non-existent. Imbued with the spirit of contributing to this field, we have been working on prodrug designs for either drugs or bioactive compounds, using many carriers, including PAMAM. Antichagasic, leishmanicide, tuberculostatic, and antimalarial PAMAM prodrugs have been designed and are being synthesized (data not published), with the purpose of improving either their pharmaceutics and pharmacokinetics, or indirectly improving their pharmacodynamic properties. As has been aforementioned in this review, the targeting of drugs mediated through PAMAM is an area that must be further explored, and we have been trying to reach better selectivity by using directing groups to achieve this. To summarize, the knowledge of all of the properties of PAMAM as a carrier, as we have briefly exposed in this review, could lead to the increase of compounds that have been submitted to clinical trials in order to introduce derivatives with better properties than their drugs/bioactive prototypes. We hope that the examples that herein have been given about PAMAM applications could incite new ideas for researchers who are involved in the fascinating world of dendrimers.
